# Relationship between GH/IGF-1 Axis, Graft Recovery, and Early Survival in Patients Undergoing Liver Transplantation

**DOI:** 10.1155/2014/240873

**Published:** 2014-04-01

**Authors:** Angela Salso, Giuseppe Tisone, Laura Tariciotti, Ilaria Lenci, Tommaso Maria Manzia, Leonardo Baiocchi

**Affiliations:** ^1^Hepatology Unit, Department of Internal Medicine, University of Rome “Tor Vergata”, Via Montpellier 1, 00133 Rome, Italy; ^2^Transplant Unit, Department of Surgery, “Tor Vergata” University, Via Montpellier 1, 00133 Rome, Italy

## Abstract

*Background*. High levels of IGF-1 have been reported in patients with initial poor function of the graft after liver transplantation (LT). Correlation with other clinical variables or early survival has not been extensively investigated. *Aim*. To evaluate the GH/IGF-1 profile as a function of liver recovery and patients' early survival after LT. *Methods*. 30 transplanted patients (23 survivors and 7 nonsurvivors), were retrospectively enrolled in the study. GH and IGF-1 serum levels were assessed at baseline, graft reperfusion, and 1, 7, 15, 30 , 90, and 360 days after LT. Individual biochemical variables were also recorded. *Results*. After grafting, IGF-1 in blood linearly correlated with cholesterol (*r* = 0.6, *P* = 0.001). IGF-1 levels from day 15 after surgery were statistically higher in survivors as compared to nonsurvivors. ROC curves analysis identified an IGF-1 cut-off >90 *μ*g/L, from day 15 after surgery, as a good predictor of survival (sensitivity 86%, specificity 95%, and *P* < 0.001). *Conclusions*. After LT, GH levels correlate with the extent of cytolysis, while IGF-1 is an indicator of liver synthetic function recovery. IGF-1 levels >90 *μ*g/L (day 15–30) seem to be an indicator of short-term survival.

## 1. Introduction

The growth hormone/insulin like growth factor-1 (GH/IGF-1) axis is of paramount importance in growth and development and is also a lifespan determinant in animal models [1–5]. IGF-1 is considered the principal mediator of GH effects in tissues. Its production is stimulated primarily in the liver through STAT 5 pathway [1, 2]. While the GH remains the major regulator of IGF-1 liver production, in turn IGF-1 counteracts GH synthesis in a classic negative feedback system. Liver diseases such as cirrhosis are characterized by a deranged GH-IGF-1 system, with increased levels of GH and reduction in IGF-1 production [6–9]. As a result patients with liver cirrhosis exhibit GH serum levels, and half-lives, that are twofold those of normal subjects. This is possibly due to reduced GH clearance and/or acquired resistance for decreased IGF-1 liver production. Reduced IGF-1 serum levels are associated with an unfavourable prognosis in cirrhotic patients [10, 11]. The reasons may be related to consequent malnutrition, insulin resistance, and immunologic impairment. In this perspective some attempts with replacement hormonal therapy have been made in liver cirrhosis showing encouraging results [12, 13]. Few studies are available on the GH-IGF-1 axis in adult patients undergoing liver transplantation (LT) [14–17]. All studies show the prompt return to normal of the GF-IGF-1 axis after transplant. One of these studies attempted to analyze the GH-IGF-1 axis trend as a function of graft recovery [18]. The authors described higher levels of IGF-1 on the first day after LT in patients with Initial Poor Function (IPF) and a normalization of IGF-1 serum levels over a longer period (*≈*3 months) in this category of subjects. Unfortunately, this interesting work, probably given the small number of patients, did not find correlations between fluctuation of GH-IGF-1 axis and other biochemical variables, and patient survival was not examined. In the present study we reassessed this issue, using a larger retrospective cohort of patients and including data on GH and IGF-1 in correlation with common biochemical variables and patient survival.

## 2. Patients and Methods

### 2.1. Patients

Thirty patients (23/7, M/F; mean age 55.2 ± 11.7) undergoing LT for various indications at our institution were retrospectively selected for this study. Exclusion criteria were (i) retransplantation; (ii) multiorgan transplantation; (iii) transplantation for fulminant hepatic failure; (iv) hepatic cirrhosis complicated by hepatocarcinoma (HCC); (v) body mass index (BMI) > 30; (vi) serum samples not available for GH and IGF-1 assessment. The protocol of the retrospective study was submitted and approved by our local ethics committee. The operation was performed according to standard technique, with the use of venovenous bypass and the positioning of a T-tube to protect the biliobiliary anastomosis. The T-tube was removed three months after grafting, in compliance with the standard protocol of our and other liver units [19, 20]. Demographic baseline features including the etiology of liver disease are included in Table 1. All patients underwent immunosuppression with either tacrolimus or cyclosporine monotherapy, and, according to our centre's protocol, no steroids or other immunosuppressants were used [21]. Ten age-matched healthy volunteers with normal liver enzymes and synthesis served as controls.

### 2.2. Serum Sampling

Blood samples were collected from each patient at listing, during LT (graft reperfusion phase), and on postoperative days 1, 7, 15, 30, 90, and 360. Plasma GH and IGF-1 levels were measured using commercially available ELISA kits (Pantec, Milan, Italy) and following the vendor's instructions. Other common biochemical parameters were assessed in the central laboratory facilities of our hospital (Policlinico di Tor Vergata, University of Rome “Tor Vergata”; Rome, Italy), daily during the first month and weekly or monthly thereafter.

### 2.3. Statistical Analysis

The statistical analysis was performed using the Student's *t*-test for paired or unpaired data and Pearson *r* for correlation analysis. Receiver operating characteristic (ROC) curves were designed to assess the sensitivity and the specificity of GH and IGF-1 serum levels in predicting survival, as previously described by our group in another study [22]. In brief, after ROC curves were constructed, the corresponding areas under the curves (AUC) were calculated. Next, the sensitivity, specificity, positive predictive value, and negative predictive value were calculated. The optimal cut-off value to predict mortality was determined using the Youden index method, which defines the cut-off in terms of the maximal sum of sensitivity and specificity. Survival analysis was conducted using the Kaplan Meier test and statistical differences were evaluated using the log-rank test as previously described [23]. All statistical analyses were conducted using the Statistical Software Package NCSS (Kaysville, UT). A *P* < 0.05 was considered statistically significant.

## 3. Results

### 3.1. Patients

Baseline characteristics of patients selected for the study are summarized in Table 1. The main reason for liver transplantation was virus related (HCV or HBV) end stage liver disease. All patients received a whole cadaveric organ. No acute rejection episode was recorded. Seven patients died within three months of liver transplantation. In all these patients the final cause of death was multiorgan failure, originating from graft dysfunction in four, graft dysfunction associated with renal insufficiency in two, liver and renal dysfunction associated with biliary tract complications in one.

### 3.2. Serum Levels of GH and IGF-1

**Figure 1:**
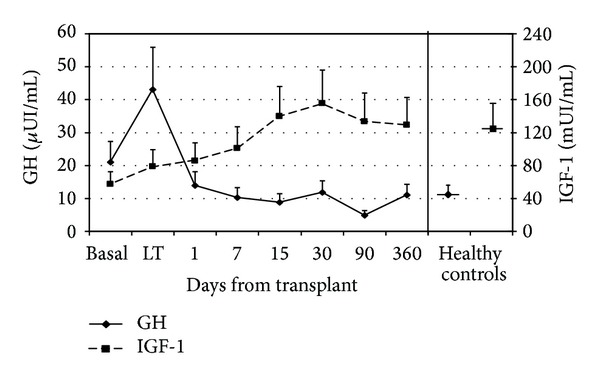
Mean values of GH and IGF-1 in the pretransplant (basal) phase, at liver transplantation (LT), and after transplant until day 360. GH and IGF-1 results are plotted according to left and right axis, respectively. Results of healthy controls (right portion of the graph) are also reported for comparison.

**Figure 2:**
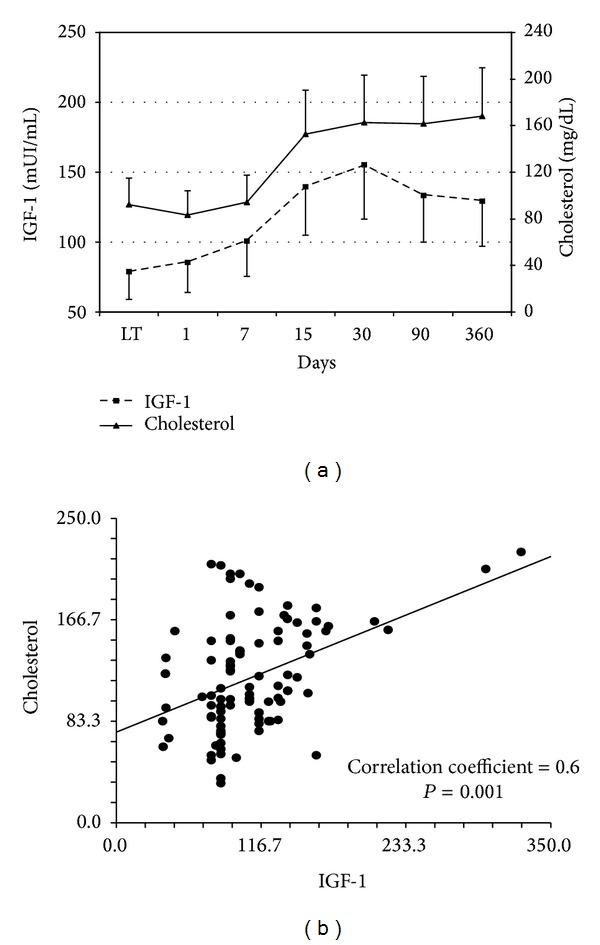
(a) Mean values of IGF-1 and cholesterol at liver transplantation (LT) and after transplant until day 360 are reported. IGF-1 and cholesterol results are plotted according to left and right axis, respectively. (b) IGF-1 and cholesterol correlation plot is depicted together with correlation coefficient and *P* value.

Mean levels of serum GH and IGF-1 at time of transplant and thereafter are shown in Figure 1 together with values of control individuals. Basal levels of GH and IGF-1 were statistically different from the control group: GH *μ*UI/mL 21 ± 7 versus 12 ± 4 in controls (*P* < 0.001) and IGF-1 mUI/mL 57 ± 16 versus 125 ± 36 in controls (*P* < 0.001). Liver transplantation determined a progressive normalization of the mean values of these two hormones. During the recovery phase after surgery IGF-1 and GH plasma levels were inversely correlated. However, this correlation was not strict (Pearson *r* = −0.2; *P* = 0.05). Correlation analysis of GH and IGF-1 with other biochemical patient variables showed a correlation between IGF-1 and cholesterol levels (Figure 2).

### 3.3. Comparison of Serum Levels of GH and IGF-1 among Survivors and Nonsurvivors

**Figure 3:**
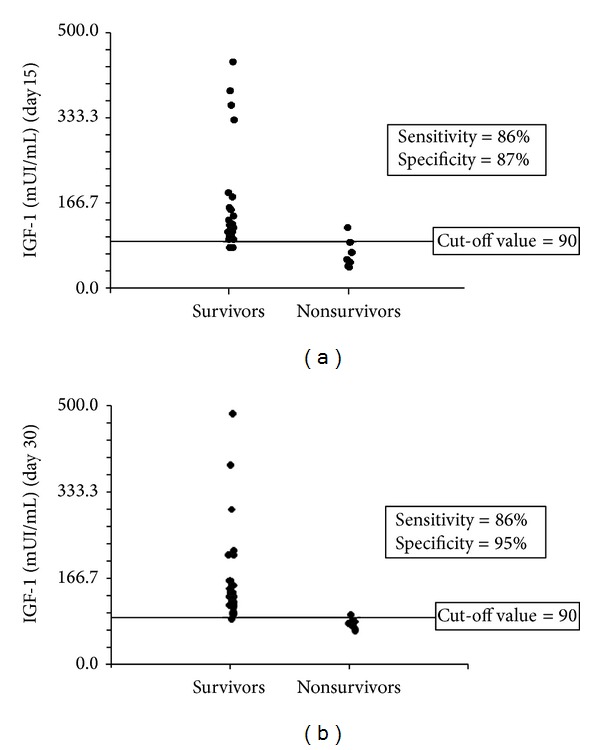
(a) Dot plot analysis of IGF-1 serum levels at day 15 after liver transplantation according to short-term (>3 months) survival or nonsurvival. Sensitivity, specificity, and cut-off value are also reported. (b) Dot plot analysis of IGF-1 serum levels at day 30 after liver transplantation according to short-term (>3 months) survival or nonsurvival. Sensitivity, specificity, and cut-off value are also reported.

**Figure 4:**
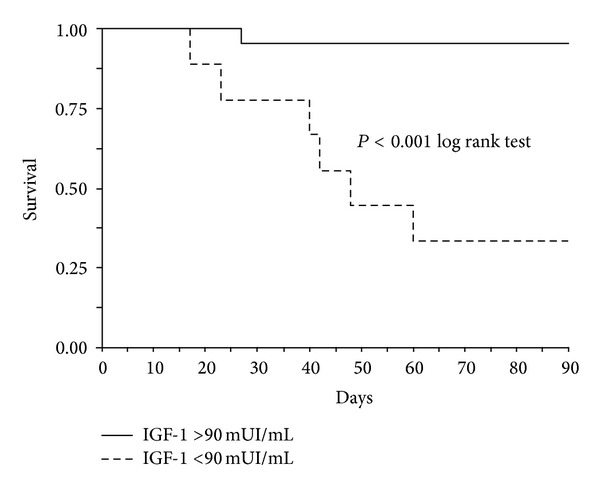
Short-term (3 months) survival analysis in patients presenting IGF-1 serum levels < (dotted line) or > (solid line) 90 mUI/mL between days 15 and 30 after liver transplant. Statistically significant difference (*P* < 0.001) by Log Rank test.

GH and IGF-1 serum levels of surviving and nonsurviving patients were compared after LT (Table 2). Serum analysis was restricted to the first month after transplant as all nonsurvivors died within 90 days of surgery. Patients with a favourable outcome exhibited lower levels of GH at day 15 and higher levels of IGF-1 at days 15 and 30 after transplantation. These differences were statistically significant (Table 2). In order to identify cut-off values for serum levels of GH (day 15) or IGF-1 (days 15–30) predictive of LT outcome, ROC curves were constructed with these two variables. For GH levels a cut-off value of 8.3 *μ*UI/mL was identified but with a very limited sensitivity and specificity (70% and 30%, resp.; AUC = 0.4). Conversely, analysis of IGF-1 data showed an unfavourable outcome in patients presenting serum levels < 90 mUI/mL at days 15 and 30. This IGF-1 cut-off value showed a sensitivity of 86% and a specificity of 87% (AUC 92%) at day 15 and a sensitivity of 86% and a specificity of 95% (AUC 96%) at day 30 in predicting patient survival. Dot plot analysis (days 15 and 30) with the corresponding cut-off value is shown in Figure 3. Survival analysis was also conducted with the same IGF-1 cut-off value of 90 mUI/mL as illustrated in Figure 4. Analysis showed a statistically significant increase in short-term (3 months) mortality in patients with IGF-1 serum levels < 90 mUI/mL between days 15 and 30 after liver transplant (*P* < 0.001 Log Rank test).

## 4. Discussion

Short-term mortality after LT remains an open issue, with minor differences between Europe and United States [24, 25]. Data from the European Liver Transplant Registry (ELTR) show that, in a ten-year follow-up, approximately half of the patients transplanted die within the first six months [24]. Prompt identification of patients with poor short-term prognosis after LT may be useful in making clinical decisions that may possibly improve the outcome for these subjects. Unfortunately, the Model for End Stage Liver Disease (MELD) and Child-Pugh scores that are efficiently used for prognostic evaluation of liver diseases and priority of graft allocation lack a significant sensitivity and specificity in the assessment of short-term prognosis in LT patients when used in the pre- or early posttransplant periods [26–28]. Simple evaluation of common biochemical indicators of liver function, such as albumin levels or coagulation function, is also not reliable, as supportive replacement therapy is frequently adopted in these patients. As a result, scoring systems not designed to evaluate posttransplant liver function, such as the RIFLE (risk of renal dysfunction, injury to the kidney, failure of the kidney, loss of kidney function, and end stage kidney disease) tailored to assess the severity of acute renal failure or Sequential Organ Failure Assessment (SOFA) designed to predict general morbidity in the intensive care unit, have a better performance [28]. In the light of this background we decided to evaluate whether GH or IGF-1 serum levels may be associated with graft recovery and patient survival after LT. IGF-1 is a hormone which is mainly produced by the hepatocytes. In this perspective the liver has a central endocrine role in the GH-IGF-1 axis [29]. IGF-1 deficiency has been well described in chronic liver diseases. The corresponding malnutrition status evidenced in cirrhosis has been suggested by some authors to be related to the limited production of IGF-1 by the diseased liver [8, 10, 29]. As regards LT a possible prognostic value of IGF-1 serum assessment after grafting has already been suggested. Bassanello and coauthors conducted a study on this issue in a limited number of patients [18]. Unfortunately, short-term mortality was not examined and the trend of IGF-1 serum levels was analysed only as a function of IPF of the graft. In this regard a statistically significant increase in IGF-1 was reported in IPF patients. We reassessed this issue in a study including a larger number of patients and with a longer follow-up. Our data firstly demonstrated a correlation between IGF-1 and cholesterol serum levels. The linear correlation between IGF-1 and cholesterol, both values showing a posttransplant progressive stepwise increase, suggests their relationship with graft functional recovery.

Given the variations of the GH/IGF-1 axis in the early phases after LT and the specific IGF-1 relationship with the graft restoring process, we evaluated the possible changes of these two hormones in short-term survivors and nonsurvivors after LT. Our data demonstrated that, already in the second week after surgery, liver related short-term survivors had decreased serum levels of IGF-1. The subsequent statistical analysis showed that patients with levels of IGF-1 persistently lower than 90 mUI/mL, after the first week, had only a 13-5% possibility of survival on the short-term. On the other hand, the same cut-off identified nonsurvivors in 86% of cases. The results were also confirmed by survival analysis. These data seem encouraging considering the scarcity of liver specific tests to apply after LT in the attempt to forecast short-term mortality. Early retransplantation, even if controversial for ethical and economical reasons, may be the sole option to improve the outcome of these patients in selected cases [30, 31]. However, in the absence of valid prognostic parameters, this clinical strategy cannot be pursued without an acceptable indication such as primary nonfunction and hepatic artery thrombosis. Even though we understand that the encouraging data from this single centre, preliminary, retrospective study need to be confirmed in larger series, our results suggest a possible strategy to gather clinical information related to liver function restoration after LT and possibly evaluate short-term survival. Whether exogenous therapeutic restoration of normal GH-IGF-1 levels may improve LT outcomes remains speculative at this stage.

## Figures and Tables

**Table 1:** Baseline characteristics of patients included in the study.

Number of patients	30
Age (years)	55.2 ± 11.7
Gender (M/F)	23/7
MELD score at transplant	16.2 ± 1.8
Donor age (years)	48.7 ± 24.8
Donor gender (M/F)	21/9
Cold ischemia time (minutes)	396 ± 112
Warm ischemia time (minutes)	42 ± 16
Cause of liver disease	12 HCV
	6 HBV
	5 Alcoholic
	2 Primary sclerosing cholangitis
	2 Autoimmune
	2 Cryptogenic
	1 Polycystic liver disease
Immunosuppression (cyclosporine-A/tacrolimus)	10/20

Continuous variables are expressed as mean ± standard deviation.

**Table 2:** Comparison of GH and IGF-1 serum levels in short-term (>3 months) survivors and nonsurvivors after liver transplantation.

Days after LT	Survivor (*n* = 23)	Nonsurvivor (*n* = 7)	Statistics
	GH		
1	13.9 ± 18	13.6 ± 10.6	n.s.
7	9.9 ± 15.1	11 ± 8.2	n.s.
15	5.8 ± 7.3	18.8 ± 27	*P* = 0.04
30	10.2 ± 16.8	20.1 ± 33.6	n.s.

	IGF-1		
1	85.2 ± 23.7	86.5 ± 27.7	n.s.
7	100.9 ± 35.7	101.5 ± 27.4	n.s.
15	162.8 ± 105.6	67.1 ± 35	*P* = 0.04
30	175.4 ± 94	79 ± 4.3	*P* = 0.03

Results are expressed as mean ± standard deviation. n.s.: difference not statistically significant.

## Abbreviations

(GH): Growth hormone
(IGF-1): Insulin like growth factor-1
(LT): Liver transplantation
(IPF): Initial poor function
(ROC): Receiver operating characteristic
(ALT): Alanine transaminase.
